# Nucleolar chromatin organization at different activities of soybean root meristematic cell nucleoli

**DOI:** 10.1007/s00709-012-0456-9

**Published:** 2012-09-26

**Authors:** Dariusz Stępiński

**Affiliations:** Department of Cytophysiology, Faculty of Biology and Environmental Protection, University of Łódź, Pomorska 141/143, 90-236 Łódź, Poland

**Keywords:** Plant nucleoli, NAMA-Ur method, Nucleolar chromatin, Chilling stress, Recovery after chilling, Electron microscopy

## Abstract

Nucleolar chromatin, including nucleolus-associated chromatin as well as active and inactive condensed ribosomal DNA (rDNA) chromatin, derives mostly from secondary constrictions known as nucleolus organizer regions containing rDNA genes on nucleolus-forming chromosomes. This chromatin may occupy different nucleolar positions being in various condensation states which may imply different rDNA transcriptional competence. Sections of nucleoli originating from root meristematic cells of soybean seedlings grown at 25 °C (the control), then subjected to chilling stress (10 °C), and next transferred again to 25 °C (the recovery) were used to measure profile areas occupied by nucleolar condensed chromatin disclosed with sodium hydroxide methylation–acetylation plus uranyl acetate technique. The biggest total area of condensed chromatin was found in the nucleoli of chilled plants, while the smallest was found in those of recovered plants in relation to the amounts of chromatin in the control nucleoli. The condensed nucleolar chromatin, in the form of different-sized and different-shaped clumps, was mainly located in fibrillar centers. One can suppose that changes of condensed rDNA chromatin amounts might be a mechanism controlling the number of transcriptionally active rDNA genes as the nucleoli of plants grown under these experimental conditions show different transcriptional activity and morphology.

## Introduction

Nucleoli are the largest subnuclear structures which are built on the basis of ribosomal genes. A nucleolus is a perfect example of an organelle where precise relationship between morphology and molecular and physiological function exists. The ultrastructure of a plant nucleolus is well known. Four main domains, fibrillar center (FC), dense fibrillar component (DFC), granular component (GC), and nucleolar vacuoles, are present, the latter only in active plant nucleoli (Stępiński 2009). The defined functions connected with ribosome biosynthesis starting from ribosomal DNA (rDNA) localization and its transcription through pre-rRNA maturation and processing to assembly of small and large ribosomal subunits have been attributed to particular nucleolar subcompartments (Raška et al. 2004).

In most eukaryotic cells, the nucleoli are formed of the final segments of chromosomes containing nucleolus organizer regions (NORs) seen as secondary constrictions on metaphase chromosomes. rRNA genes which are arranged in tandem repeated arrays are localized in them. The number of rDNA genes is species-specific. Plant cells usually possess greater number of ribosomal genes, up to several thousands, and these genes are thought to represent a greater part of nucleolar chromatin than in animal cells where only several hundreds of rRNA genes are present as it is in human (Long and Dawid 1980). Only a part of all rDNA gene pool is transcribed, while the other is inaccessible to psoralen and transcriptionally incompetent. The ratio of active and inactive rDNA gene copies can vary depending among others on species, cell type, and age (Conconi et al. 1989). Transcriptional competence of ribosomal genes as well as nucleolar dominance is governed by epigenetic mechanisms (Lawrence et al. 2004). In addition to active and silent genes, there is also a poised rRNA gene state which is between the former two (Németh and Längst 2011). It is accepted that plant ribosomal chromatin functionally related to nucleolus derives from nucleolus-associated chromatin (NAC), large condensed chromatin blocks. It usually accompanies a nucleolus and is located to its surface that frequently contains centromeric and pericentromeric chromosomal regions (Motte et al. 1991). rDNA chromatin forms higher order structures depending on r-gene activity and localizes to different subnucleolar regions (Bassy et al. 2000; Wei et al. 2002).

Although the structure and function of nucleoli and nucleolar chromatin have been extensively studied for decades in both plant and animal systems using different light and electron microscope techniques including 3D scanning visualization (Iwano et al. 2003; Kodiha et al. 2011), their new functions and precise molecular mechanisms associated with higher order organization of nucleolar chromatin are still being discovered (Motte et al. 1988; Boisvert et al. 2007; Németh and Längst 2011).

The basic principles of nucleolar chromatin organization seem to be general for higher plants as the distribution and arrangement of the ribosomal chromatin were similar for both mono- and dicotyledonous species (Motte et al. 1991). Nucleolar rDNA is mainly located in FCs and in DFC near FCs. Two types of FCs appear in the nucleoli of plant root meristematic cells: homogenous FCs, which are present in active nucleoli and are characterized by the presence of decondensed chromatin, and heterogenous FCs mainly in inactive or low active nucleoli, containing both decondensed and condensed chromatin (Risueño et al. 1982). Sometimes spots of condensed chromatin could be seen in the other regions of the nucleoli, but this chromatin could correspond to interdigitation of extranucleolar condensed chromatin into nucleolar interior (Martin et al. 1989). In addition to rDNA chromatin, a nucleolus also contains other DNA (Németh and Längst 2011).

The nucleoli of root meristematic cells of soybean seedlings grown under various temperature conditions show different transcriptional activity: lowest under chilling (10 °C), while highest during recovery at optimal temperature (25 °C), even in comparison to the control, i.e., the plants grown permanently at 25 °C (Stępiński and Kwiatkowska 2003; Stępiński 2004). The nucleolar morphology also differs under those conditions (Stępiński 2009, 2010). A presumption has been made that the changes of nucleolar transcriptional activity might result from inactivation of rRNA gene copies (chilling stress) or their activation (recovery) caused by rDNA chromatin condensation or decondensation, respectively. Hence, it was decided to measure and analyze the areas occupied by condensed nucleolar chromatin using sections of root meristematic cell nucleoli from soybean seedlings grown under the above-mentioned conditions with the use of sodium hydroxide methylation–acetylation plus uranyl acetate (NAMA-Ur), a method disclosing nuclear chromatin.

There are a number of methods to investigate nuclear chromatin and its remodeling. The chromatin immunoprecipitation (ChIP) technique as well as its variations is used to examine the interaction between DNA and proteins. It allows an estimation of the location and density of cytosine methylation, posttranslational histone modification variants, or transcriptional factors as well as enables determination of their coexistence on any genomic (e.g., euchromatic or heterochromatic) regions. The psoralen cross-linking method uses the covalent binding of DNA in chromatin to psoralen. Micrococcal nuclease (MNase) action is a method which uses different sensitivities of DNA regions being in various conformations to digestion by the MNase. With these two techniques, the chromatin competence state can be established including the proportion of transcriptionally active and inactive rRNA gene copies. Nuclear location and semi-quantitative studies of chromatin condensation state can be carried out by means of conventional fluorescent and confocal microscopy and of antibodies to markers of transcriptionally competent and incompetent chromatin, i.e., suitable modified core histones as well as to methylated cytosines in DNA.

Electron microscopy-based techniques are the alternative. The conventional method for electron microscopy (EM) does not allow to demonstrate nucleolar DNA clearly because it is masked by electron dense fibrillar and granular components. The NAMA-Ur method is a DNA-specific cytochemical staining technique which allows to display both extranucleolar nucleoplasmic DNA and nucleolar DNA in situ. In this technique, RNA is digested disclosing DNA in the nucleoli, while other cellular components are hardly distinguishable because they got bleached. DNA shows high electron density so it is clearly seen as dark clumps, blocks, or fibers in the whole nuclei including the nucleoli.

Typical biochemical methods give more precise quantitative results and are sufficient from a physiological point of view, but they do not answer the question about localization of a given chromatin type in a nucleus, whereas the NAMA-Ur electron microscopy technique is useful not merely to investigate chromatin but its additional advantage is the ability to specify the relationship between chromatin organization and other nuclear structures in situ.

## Material and methods

### Plant material and growth conditions

Soybean seeds (*Glycine max* (L.) Merr.) var. Aldana (obtained from the Plant Breeding and Acclimatization Institute in Strzelce, Poland) were germinated in darkness at 25 °C (control) on distilled water-moistened filter paper for 3 days. These 3-day-old seedlings were transferred to 10 °C for 4 days. Four-day-chilled seedlings were then recovered for 2 days at optimal temperature (25 °C). Root meristem tips of the seedlings were subsequently subjected to electron microscopy analyses.

### Conventional thin section electron microscopy

Selected root tips of plants from three experimental variants (control, chilling stress, recovery) were fixed in 2 % glutaraldehyde in 1 % cacodylate buffer (pH 7.2–7.4) for 3 h at 4 °C. The roots were postfixed in 1 % osmium tetroxide in the same buffer. After dehydration through ethanol series, the material was embedded in the medium containing the mixture of Epon 812 and Spurr’s resin. Ultrathin sections (60–80 nm) were doubly stained with uranyl acetate and lead citrate according to Reynolds (1963). The sections were examined and photographed in a JOEL JEM 1010 transmission electron microscope.

### NAMA-Ur procedure

DNA was stained with the method developed by Testillano et al. (1991) with some modifications. Briefly, the root tips were fixed in 3 % glutaraldehyde and 4 % formaldehyde in 0.1 M phosphate buffer saline (pH 7.3) for 3.5 h at 4 °C. After three washes in the same buffer (20 min each), the material was postfixed in 0.5 M NaOH and 4 % formaldehyde overnight. Then, the roots were rinsed three times in distilled water for 10 min each, then in 1 % acetic acid three times for 10 min each, and again three times in distilled water for 10 min each. Dehydration was performed in methanol at increasing concentrations of 10, 30, 50, 70, 80, 90, and 100 % twice for 15 min each. After that, the roots were treated with the freshly prepared solution of methanol–acetic anhydride (5:1, *v*/*v*) for 36 h at room temperature until the roots were properly bleached. After the material was washed in pure methanol twice for 20 min each, it was treated with propylene oxide twice for 10 min and then impregnated with the mixture of propylene oxide and Epon 812 in 2:1 and 1:1 proportions for 3 h each, then in 1:2 proportion through the night. Finally, the root tips were embedded in Epon 812 resin and polymerized at 37, 60, and 70 °C for 24 h each. Semi-thin sections (140 nm) were stained with 5 % aqueous uranyl acetate for 70 min at 60 °C and then additionally stained with uranyl acetate and lead citrate according to Reynolds (1963).

### Calculations and statistics

Three apical root meristems were analyzed for each variant. The nucleoli from the cortical meristematic cells in G2 phase from the division zone were chosen. Fifty microphotographs for each meristem were made. The nucleoli with the biggest sections, most probably with central sections, were chosen for analyses in order to avoid tangential sections. Images were scanned, and then at properly high magnification visible on a screen, the borders of condensed chromatin clump profiles were precisely manually drawn with a computer mouse. Next, morphometric measurements of the areas [μm^2^] of nucleoli and of nucleolar chromatin clumps were made with the use of a computer software for cytophotometry (Cytophotometr v1.2; Forel, Łódź, Poland; the software formed for our department needs only).

Mean values of condensed chromatin clump areas and standard deviations (±SD) were calculated by means of Microsoft Excel spreadsheet. Statistical significance of differences between values representing areas of the total condensed nucleolar chromatin per nucleolus in particular experimental treatments was estimated using Student’s *t* test (*p* < 0.05) and STATISTICA 8.0 Inc. (USA) computer software. The statistical sample included 50 independent measurements for each treatment.

## Results

### Conventional electron microscopy images

**Fig. 1:**
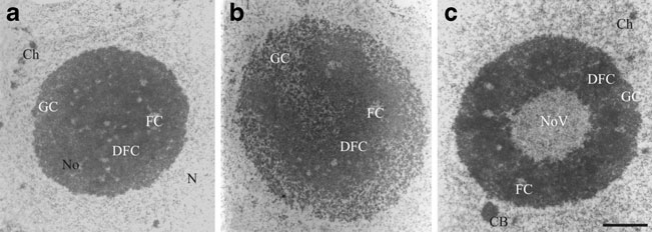
Nucleoli of root meristematic cells of soybean seedlings grown at 25 °C (control; **a**), under chilling stress (10 °C; **b**), and after recovery at 25 °C (**c**). Conventional electron microscopy procedure. *N* extranucleolar nucleoplasm, *No* nucleolus, *FC* fibrillar center, *DFC* dense fibrillar component, *GC* granular component, *NoV* nucleolar vacuole, *Ch* condensed chromatin, *CB* coiled (Cajal) body. *Bar* = 1 μm

The ultrastructure of soybean root meristematic nucleoli displayed the following clearly distinguishable areas: small, rather circular regions with the lowest electron density, FCs; around these areas, the DFC with the highest electron density; GC was located at the peripheral part of the nucleoli and around DFC; and nucleolar vacuoles (NoV) could be mainly seen in the nucleoli with high transcriptional activity. The metabolic activity of soybean nucleoli is correlated with their morphology which changes together with the experimental treatment applied: the control nucleoli (Fig. 1a)—middle activity; nucleoli of stressed plants (Fig. 1b)—low activity; and nucleoli of recovered plants (Fig. 1c)—high activity. Detailed description of these diversities one can find in earlier papers (Stępiński 2009; Stępiński 2010). Nucleolar chromatin is poorly visible in these micrographs because of high electron density of nucleolonema which masks chromatin which also displays high electron density. In order to directly compare nucleolar structures observed after NAMA-Ur or conventional EM fixation, both procedures were performed on the same specimens, on soybean root meristematic cells.

### NAMA-Ur technique images

**Fig. 2:**
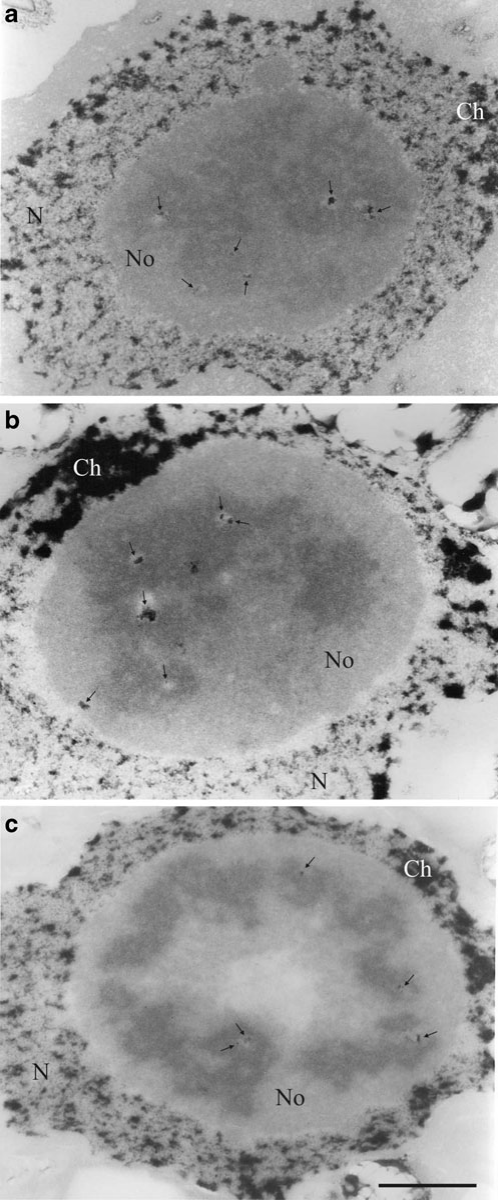
Nucleoli of root meristematic cells of soybean seedlings grown at 25 °C (control; **a**), under chilling stress (10 °C; **b**), and after recovery at 25 °C (**c**). NAMA-Ur technique staining of chromatin. *N* extranucleolar nucleoplasm, *No* nucleolus, *Ch* condensed chromatin, *arrows* point to condensed chromatin clumps localized mainly to FCs. *Bar* = 1 μm

The NAMA-Ur cytochemical technique specifically uncovers the ultrastructure of DNA; thus, chromatin is revealed as highly contrasted, dark areas both at the extranucleolar nucleoplasm and nucleolar territories. In the nucleoplasm, irregular clumps of condensed chromatin, frequently near the nuclear envelope as well as at the nucleolar periphery probably as nucleolus-associated chromatin, were darkest stained (Fig. 2a–c).

**Fig. 3:**
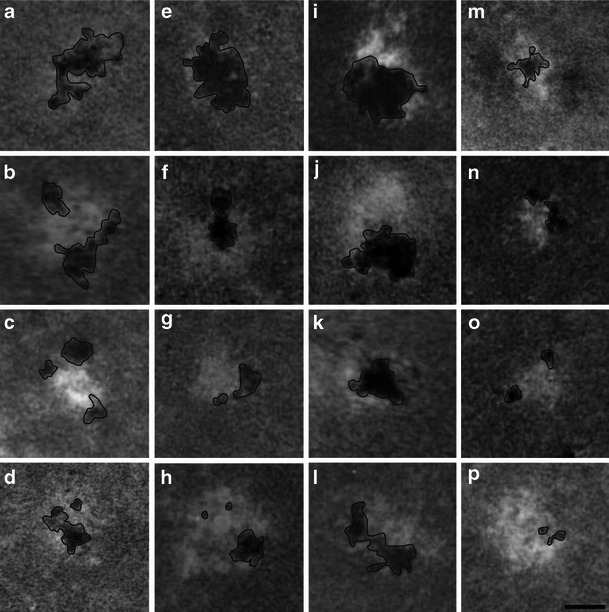
Examples of shape patterns (**a**–**h**) and sizes, from the biggest to the smallest (**i**–**p**), of condensed chromatin clumps localized to FCs of nucleoli of soybean root meristematic cells. NAMA-Ur technique staining of chromatin. The areas that were subjected to measurements are outlined with a *black line*. *Bar* = 100 nm

At the nucleolar territories, the chromatin was located mainly in the FC regions, rarely in DFC or GC in all three experimental variants. The total area occupied by single condensed chromatin clumps visible on nucleolar sections varied between particular experimental variants. The biggest total area of chromatin was observed in the nucleoli of plants subjected to chilling stress (Fig. 2b, Table 1), while the smallest was found in those from the recovered plants (Fig. 2c, Table 1), and the control values were in between (Fig. 2a, Table 1). The differences between chromatin areas resulted rather from various sizes of single heterochromatic areas placed mainly in FCs (Fig. 3a–p) than from various numbers of condensed chromatin clumps. The biggest single chromatin areas were observed in FCs of the nucleoli of the chilled plants (Fig. 2b), while the smallest were in those of the recovered seedlings (Fig. 2c). In the control, nucleoli heterochromatic areas were of intermediate sizes (Fig. 2a). The masses of condensed chromatin were mainly located in the peripheral territory of FCs, rarely in their central parts (Fig. 3a–p). The clumps of chromatin were of various shapes and their numbers in single FC ranged from one to three. The clumps were of various sizes, from small which occupied tiny areas of FC to large ones, about 100-fold bigger, which occupied whole FCs (Fig. 3a–p).

**Table 1:** Area [μm^2^] of condensed chromatin per nucleolus estimated in cross-sectioned nucleoli of soybean root meristematic cells in plants grown under the control, chilling, and recovery conditions

Experimental treatment	Area	
	Nucleolar condensed chromatin [μm^2^]	Nucleolus [μm^2^]
Control	0.11 ± 0.02	8.4 ± 0.9
Chilling stress	0.25 ± 0.03	11.2 ± 1.3
Recovery	0.08 ± 0.002	14.0 ± 1.5

There were statistically significant differences concerning the areas of nucleolar chromatin between values estimated for the control and the chilling stress and between the control and the recovery, *p* < 0.05

Dark areas probably corresponding to DNA were also present in the DFC and GC territories. This chromatin could be located in nucleolar channels and continuous with perinucleolar chromatin. These channels are not always clearly seen depending on the section obtained.

## Discussion

NAMA-Ur, the method displaying nuclear chromatin in electron microscopy, allowed to identify indubitably the areas occupied by condensed chromatin in the nucleoli of soybean root meristematic cells. Modification of this method consisting of extra staining of the sections on grids according to Reynolds (1963) disclosed darker areas inside nucleolar territories probably corresponding to nucleolonema which is composed of DFC and FCs. A classic electron microscopy does not allow to disclose unequivocally chromatin clumps in the nucleolar territory in soybean plants (Stępiński 2010). After the use of the NAMA-Ur technique, it turned out that soybean nucleolar FCs did contain the clumps of condensed chromatin.

In soybean nucleoli, chromatin was mainly located in the periphery of FC, and rarely, it was seen in their interior. This chromatin was in the form of condensed state. Nucleolar chromatin may be located in different positions and its condensation state may vary which may imply different status of rDNA transcription in various plant species (Long et al. 2008). In *Zea mays* and *Salix alba*, root meristematic cell nucleoli DNA was localized almost exclusively in FCs (Motte et al. 1991). In *Hordeum vulgare* (barley), root meristem nucleoli DNA was detected only at the periphery of FCs (Iwano et al. 2003). *Vicia faba* nucleolar DNA was distributed in the FCs, at the periphery of FCs, and at the FC/DFC boundaries. In this case, extended DNA fibers in the center of FCs, condensed DNA clumps at the FC periphery, and at the FC/DFC border in semi-circular or crescent formations were found (Long et al. 2004). Nucleolar chromatin can be also found in the DFC territory far from FCs. Most probably, it corresponds to interdigitation of extranucleolar condensed chromatin into the nucleolar body (Martin et al. 1989). rDNA was even detected in nucleolar vacuoles of large nucleoli of pea. It is suggested that most of the active rDNA genes are widely dispersed throughout the nucleolus in small foci (Shaw et al. 1996). However, most frequently, plant nucleolar DNA is localized inside FCs, in two forms, as condensed and dispersed chromatin, and at the border of FC and DFC in the form of irregular clumps.

Chromatin in soybean nucleoli, as can be seen in the micrographs of nucleolar sections, occupies a relatively small area. This is probably species specific because the nuclei of soybean root meristematic cells belong to euchromocentric type of nuclei in which only chromocenters are seen in fluorescence microscopy after DAPI staining (Shi et al. 1996), with a small amount of DNA, ca. 2.43 pg per 2C DNA (Chung et al. 1998). These nucleoli contain a relatively small number of rDNA genes, about 2,000 copies (Chen et al. 1983), as only one chromosome contains NOR (Yang and Jeong 2008), forming one nucleolus, and a single locus for 45S rRNA genes exists (Shi et al. 1996). That is why so little amounts of chromatin are detected in soybean nucleoli, and chromatin location is limited mainly in FCs. In DFC and GC territories, the chromatin is seldom detected depending on the section. Other species, for instance, onion root meristematic cell nuclei, contain much more DNA, ca. 33.55 pg per 2C DNA (Van’t Hof 1965) and contain about 7,000 copies of rRNA genes grouped in five clusters which form two nucleoli at the most (Panzera et al. 1996), that is why onion nucleolar chromatin is abundantly seen in homogeneous and heterogenous FCs, in DFC near FCs as well as in DFC itself (Martin et al. 1989; González-Melendi et al. 1998; Tao et al*.* 2001b).

Measurements of areas occupied by nucleolar chromatin in soybean cells of three experimental treatments (control, chilling stress, recovery) showed that the amount of condensed chromatin could change in the nucleoli and was correlated with the temperature of plant growth—masses of condensed chromatin increased during the chilling treatment, while decreased when plants were transferred to the optimal temperature for recovery. It can be supposed that condensation and decondensation of chromatin make a mechanism controlling the number of active rDNA genes depending on the need for machineries producing proteins—ribosomes. During unfavorable conditions, under chilling, when soybean plants show considerably reduced growth and development, a great number of active rRNA genes are not required, while during recovery when plants want to make up for losses caused by stress, the extra rRNA genes could be activated. The results of earlier autoradiographic studies with the use of ^3^ H-uridine which showed that incorporation of the radioactive transcription precursor was considerably reduced under chilling, while during recovery it was even higher than in the control plants seem to confirm this hypothesis (Stępinski and Kwiatkowska 2003; Stępiński 2004).

The activity of ribosomal RNA genes can be regulated by suppression of the whole locus (all tandemly arranged genes) or by suppression of only some genes within a single locus which can be achieved by increased DNA methylation (Ellis et al. 1990) resulting in chromatin condensation. It is generally believed that nucleolar transcriptional activity is correlated with rDNA transition from condensed, inactive rDNA chromatin to loosened, transcriptionally competent rDNA chromatin, and back. In addition, rRNA genes exist not only as active and silent genes, but also as poised ones. These are in chromatin state between active and inactive genes. It is thought that poised, nontranscribed genes are located in FCs, while after they have been activated, they move towards the FC and DFC border (McKeown and Shaw 2009; Németh and Längst 2011). In order to increase transcriptional efficiency in plant nucleoli, the compacted chromatin of FCs gradually disperses into small fragments or into fine fibrils turning transcriptionally incompetent chromatin into a competent one (Highett et al. 1993). It is suggested that transcriptionally inactive chromatin being in decondensed state, which is present together with condensed also fully inactive chromatin, in FCs at the given time, as it is in onion plants, may be ready for rRNA transcription when it is required (Risueño et al. 1982). In plants, nucleolar chromatin located in the peripheral parts of FCs and also in the border between FC and DFC is most probably associated with transcriptionally active rDNA as at these regions the low concentrated, extended rDNA fibers overlap with the site of rRNA transcription, while the dense chromatin usually present in the middle of FCs is associated with inactive rDNA chromatin (Yano and Sato 2000; Tao et al. 2001a, 2002). It is believed that condensed rDNA chromatin in FCs, usually transcriptionally inactive, makes a reservoir of rRNA genes. Such situations could be referred to the events appearing in soybean plants subjected to experimental treatments presented currently. Most nucleolar chromatin in soybean nucleoli in all experimental variants was identified at FC peripheries. Such location may suggest that condensation and decondensation take place at this region during the chilling treatment and recovery, respectively.

The changes in the amount, location, and condensation state of nucleolar chromatin were also observed in other plant species under different stress conditions. In *Pisum sativum*, root meristematic cell active nucleoli DNA was located in DFC by means of terminal deoxynucleotidyl transferase (TdT) immunogold method, in which non-isotopic deoxynucleotides are added to 3′-ends of DNA by specific DNA polymerase. Whereas in nucleoli inactivated with low temperature, no significant labeling was observed in DFC, but intense label was seen over condensed chromatin in large heterogeneous FCs (Mineur et al. 1998). The authors suggest that in plant cells, inactivation of rRNA genes is accompanied by changes in the conformation of ribosomal chromatin and the presence of DNA in DFC depends on the level of rRNA gene activity. When *V. faba* roots were exposed to hypoxia stress, DNA of DFC around FCs, site of rRNA transcription, temporarily formed condensed chromatin fragments in the FCs (Yamada and Sato 1996). Also in *Lepidium sativum* (cress) root meristematic cells grown under clinorotation (simulated microgravity), a kind of stress for plants, the amount of condensed DNA localized in FCs was much greater than that of noncondensed DNA present in the FC–DFC transition zone in comparison to the control conditions. Thus, re-localization of nucleolar DNA from DFC and the FC–DFC transition zone to FCs occurred. rDNA transcription was also reduced under clinorotation since some of rDNA switched from active into inactive state (Sobol et al. 2005).

The results of the studies obtained recently by means of fluorescence microscopy and antibodies to euchromatin and heterochromatin markers, i.e., methylated or acetylated lysine residues on core histones as well as methylated cytosine in DNA, are the support of the current work. It was shown that the nuclear chromatin state changed in a way similar to that described in this work, i.e., the amount of condensed chromatin increased when soybean seedlings are transferred from optimal temperature to chilling, whereas it decreased under recovery conditions (Stępiński 2012).

Taken together, the exact arrangement, distribution, and localization of nucleolar DNA depend on cell type, cell cycle phase, species, metabolic activity, and growth conditions (Long et al. 2008; Shang et al. 2009). Different locations as well as condensation states of nucleolar DNA may imply various status of transcriptional competence of rDNA. It seems that in plant cells, the decrease in rRNA transcription under unfavorable conditions may lead to the reduction of r-gene number through condensation of ribosomal chromatin.
